# Hal2p Functions in Bdf1p-Involved Salt Stress Response in *Saccharomyces cerevisiae*

**DOI:** 10.1371/journal.pone.0062110

**Published:** 2013-04-17

**Authors:** Lei Chen, Liangyu Liu, Mingpeng Wang, Jiafang Fu, Zhaojie Zhang, Jin Hou, Xiaoming Bao

**Affiliations:** 1 State Key Laboratory of Microbial Technology, Shandong University, Jinan, China; 2 Department of Zoology and Physiology, University of Wyoming, Laramie, Wyoming, United States of America; Texas A&M University, United States of America

## Abstract

The *Saccharomyces cerevisiae* Bdf1p associates with the basal transcription complexes TFIID and acts as a transcriptional regulator. Lack of Bdf1p is salt sensitive and displays abnormal mitochondrial function. The nucleotidase Hal2p detoxifies the toxic compound 3′ -phosphoadenosine-5′-phosphate (pAp), which blocks the biosynthesis of methionine. Hal2p is also a target of high concentration of Na^+^. Here, we reported that *HAL2* overexpression recovered the salt stress sensitivity of *bdf1Δ*. Further evidence demonstrated that *HAL2* expression was regulated indirectly by Bdf1p. The salt stress response mechanisms mediated by Bdf1p and Hal2p were different. Unlike *hal2Δ*, high Na^+^ or Li^+^ stress did not cause pAp accumulation in *bdf1Δ* and methionine supplementation did not recover its salt sensitivity. *HAL2* overexpression in *bdf1Δ* reduced ROS level and improved mitochondrial function, but not respiration. Further analyses suggested that autophagy was apparently defective in *bdf1Δ*, and autophagy stimulated by Hal2p may play an important role in recovering mitochondrial functions and Na^+^ sensitivity of *bdf1Δ*. Our findings shed new light towards our understanding about the molecular mechanism of Bdf1p-involved salt stress response in budding yeast.

## Introduction

Like many other organisms, the budding yeast Saccharomyces cerevisiae possesses multiple mechanisms to adapt to various environmental stresses. When yeast encounters salt stress, for instance, the plasma membrane Na^+^-ATPase, encoded by ENA1/PMR2, pumps excess Na^+^ to outside of the cell [1]. Other pathways, like HOG-MAPK pathway and calcineurin signaling pathway may also be activated by salt stress [2]. In addition, many other genes, including NHA1 (encoding a Na^+^/H^+^ antiporter located at the plasma membrane), TRK1/TRK2 (encoding K^+^ transporters located at the plasma membrane), and NHX1 (encoding a Na^+^/H^+^ antiporter localized in the cytoplasm), also participate in salt stress response in yeast [3], [4].

Bromodomain Factor 1 (Bdf1p) belongs to the bromodomain and extra-terminal (BET) family, a novel group of transcriptional regulators [5]–[8]. Bdf1p contains two copies of the bromodomain, which binds to the acetylated lysine residue(s) of histone H3 or H4 [9]–[11]. It also contains one copy of the extra-terminal (ET) domain, which can be phosphorylated by casein kinase 2 (CK-2) [7]–[8]. Bdf1p associates with the basal transcription complexes TFIID and corresponds to the carboxyl-terminal region of TAF_II_250 [12]. Bdf1p is also a member of the SWR1 complex, which is required for recruitment of histone H2A variant htz1 onto chromatin [13].

Previous studies have shown that *BDF1* plays a role in multiple stresses, including salt, high temperature, caffeine and LiCl [14]. Our previous data demonstrate that *BDF1* deletion causes mitochondria dysfunction and apoptosis under salt stress; and the Bdf1p-involved salt stress response is independent of Ena1p, Trk1p, MAPK pathway and calcineurin signaling pathway [15]. However, the molecular mechanism of Bdf1p-involved salt stress response remains unclear.

*HAL2* (also named as MET22) encodes a bisphosphate-3′-nucleotidase, which converts toxic 3′ -phosphoadenosine-5′-phosphate (pAp), the intermediate product of the sulfate assimilation pathway, into nontoxic AMP and Pi. Hal2p is inhibited by high concentration of Na^+^ or Li^+^, leading to pAp accumulation [16]. The accumulated pAp then inhibits the 5′-3′-exoribonuclease activity and blocks the biosynthesis of methionine [17]–[19].

In this study, we revealed that overexpression of *HAL2* increased the salt resistance of *bdf1Δ*. We further demonstrated *HAL2* expression was regulated by Bdf1p. Further analysis suggests that Hal2p may enhance *bdf1Δ* salt resistance by stimulating autophagy, which removes harmful substances, such as reactive oxygen species (ROS) in *bdf1Δ*.

## Materials and Methods

### Plasmids and Strains Construction

All plasmids and the S. cerevisiae strains used in this study were listed in Table 1. *HAL2* or *BDF1* ORF was cloned into a 2-µm plasmid pYX242, resulting in pYX242-*HAL2* or pYX242-*BDF1*, respectively. GFP-*ATG8* was cloned into plasmid pRS316 [20], named pRS316-GFP-*ATG8*. The plasmids were transformed into different strains as described in the results section. The DNA fragments of the disruption cassettes for the target genes were amplified by four primers [21] and transformed into W303-1A or *bdf1Δ* [15], producing different deletion strains. The deletion strains were verified by PCR. The primers used for PCR amplification were listed in Table S1. *BDF1*-Flag strain was constructed as previously described [22].

**Table 1:** Strains and plasmids used in this study.

Strains	Genotype/Properties	Reference/Resource
*Saccharomyces cerevisiae* W303	*MATa, leu2-3/112 ura3-1 trp1-1 his3-11/15 ade2-1 can1-100*	[14]
*bdf1Δ*	Derivative of W303: *BDF1*::kanMX4	This study
W303+pYX242	W303 transformed with empty pYX242	This study
*bdf1Δ*+pYX242	*bdf1Δ* transformed with empty pYX242	This study
*bdf1Δ*+*HAL2*	*bdf1Δ* transformed with pYX242-*HAL2*	This study
*bdf1Δ*+*BDF1*	*bdf1Δ* transformed with pYX242-*BDF1*	This study
W303+*HAL2*	W303-1A transformed with pYX242-*HAL2*	This study
*ena1Δ*	Derivative of W303-1A: *ENA1*::*HIS3*	[32]
*hal2*Δ	Derivative of W303-1A: *HAL2*::*URA3*	This study
*bdf1Δhal2*Δ	Derivative of *bdf1Δ*: *HAL2*::*URA3*	This study
*BDF1*-Flag	Derivative of W303-1A: *BDF1*::*BDF1*–Flag	Deposited in our lab
plasmids		
pRS316-GFP-*ATG8*	GFP-*ATG8* fusion segments cloned to pRS316	This study
pRS315-GFP-*ATG8*	GFP-*ATG8* fusion segments cloned to pRS315	This study
pYX242+*BDF1*	*BDF1* ORF segments cloned to pYX242	This study
pYX242+*HAL2*	*HAL2* ORF segments cloned to pYX242	This study

### Spot dilution growth assay

The growth phenotype of the strains was analyzed by spot assay as previously described [23]. Briefly, cells were cultured overnight either in YPD media (1% yeast extract, 2% peptone, 2% glucose) or in complete synthetic defined (SD) medium (0.17% yeast nitrogen base, 0.5% (NH_4_)_2_SO_4_, 2% glucose, and supplemented with arginine, histidine, tryptophan, uracil, adenine and leucine). Cells were then harvested by centrifugation. Cells were washed twice and resuspended in ddH_2_O. The cell density was normalized to an OD_600_ = 1.0. Four µl of each ten-fold serial dilution of the cultures were spotted onto appropriate solid medium. Growth was monitored after 3 days at 30°C.

### Na^+^ or Li^+^ treatment

The overnight cultures were inoculated in 100 ml fresh medium and then grown to mid-log phase at 30°C. Half of the culture was transferred to media containing final concentration of 0.5 mol.L^−1^ NaCl or 0.1 mol.L−1 LiCl. Cells were grown at 30°C for 45 min for most of the experiments, except for concentration detection of Na+ (5 h) or pAp (4 h with NaCl or 2 h with LiCl). Samples without salt treatment were used as controls in all cases.

### Determination of Na^+^ and pAp concentration

To detect the intracellular Na^+^ concentration, cells were washed four times with 20 mmol.L^−1^ MgCl_2_. The air-dried cells were nitrified in a nitrification tube with 3 ml nitric acid for 1 hour at room temperature. Five ml of ultra-pure water was then added to the nitrified cells and incubated in a Microwave Digestion System (Milestone) for 45 min at 120°C. The Na^+^ concentration in the nitrified cells was analyzed by atomic absorption spectrophotometry (SOLAAR) at 589 nm with air-acetylene flame and 1.1 L.min^−1^ gas flows [6], [24], [25].

To detect the intracellular pAp concentration, salt-treated cells were harvested and extracted with 1 ml of 2 mol.L^−1^ perchloric acid in a ice-bath for 15 min. Extracts were centrifuged at 2,000 rpm for 5 min. 900 µl supernatant was adjusted to pH 6.0. After centrifugation, the supernatant was filtered by filter membrane for HPLC analysis. Yeast nucleotide extraction and HPLC analysis were conducted as described previously [26]. 10 µl of each extract were analyzed by HPLC (SHIMADZU). Samples were applied to a reversed phase C18 column (5-µm particle size, GL Sciences), eluted and detected as described previously [26]. Nucleotide peaks were identified by co-injection with standards (AMP, ADP, ATP and pAp from Sigma).

### RNA extraction and real-time quantitative PCR (RT-qPCR)

The treated cells were rapidly frozen by liquid nitrogen. Total yeast RNA was isolated using UNIQ-10 spin column RNA purification kits (BBI) in accordance with the manufacturer's instruction. Total RNA was treated with DNase I (Takara) to eliminate genomic DNA. 2 µg of DNA-free total RNA was used to synthesize the first strand of cDNA in 20 µl reverse transcription (RT).One µl of RT reaction product was used for qPCR using the LightCycle PCR System (Bio-Rad) and SYBR Green I monitoring method. The forward and reverse specific primers were listed in Table S1. The ACT1 gene was used as a reference for normalization. Fold changes in gene expression were calculated using the comparative 2^−ΔΔt^ method [5].

### Western blot

Protein sample preparation, SDS-PAGE and Western blots were performed as described previously [27]. Primary antibodies were polyclonal rabbit anti-yeast Hal2p, anti-β-tubulin (ANBO) and anti-rabbit IgG (H+L) (ZSGB, ZB2301).

### Chromatin immunoprecipitation (ChIP)

ChIP was accomplished using ENZ ChIP kits (MILLIPORE) according to the manufacturer's instruction. Yeast cell wall was removed by zymolase as described previously [28]. Immunoprecipitation was performed with the following antibodies: rabbit anti-Flag (sc-807; Santa Cruz Biotechnology) and normal rabbit IgG (sc-2027; Santa Cruz Biotechnology). Primers used in ChIP amplifications were listed in Table S1.

### Assessment of ROS, mitochondrial membrane potential (Δ*φ*) and GFP-*ATG8*

Detection of ROS and assessment of mitochondrial membrane potential (*Δφ*) were performed as described previously [15], [29]. The values of ROS, *Δφ* and GFP-*ATG8* fluorescence were quantified as the relative fluorescence intensity using ImageJ software. Photographs were representatives of three independent experiments.

### Fluorescence microscopy

Fluorescence microscopy was performed using a Nikon ECLIPSE 80i system equipped with a plan Apochromat 40× objective (NA = 0.95) and a plan Apochromat 60× oil objective (NA = 1.40). Images were acquired and analyzed using NIS-Elements AR 3.1 software.

### Statistical Analysis of Data

A two-tailed t test, assuming equal variances, was used to determine whether the differences between the strains' behavior were statistically significant.

## Results

### 1. The high sensitivity of *bdf1Δ* to salt stress was not caused by intracellular Na^+^ accumulation

**Figure 1:**
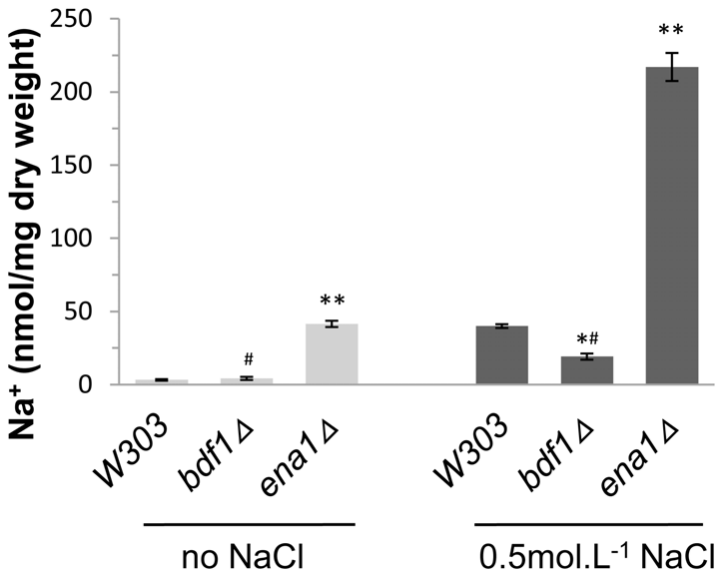
The intracellular Na+ concentration in *bdf1Δ* was lower than that in wild type. Mid-log phase cells were grown for 5 h with or without 0.5 mol.L^−1^ NaCl. The treated cells were washed with MgCl_2_ and air dried. The dried cells were nitrified with nitric acid. The Na^+^ concentration was analyzed by atomic absorption spectrophotometry at 589 nm. Error bars denote standard deviation (SD). *P<0.05, **P<0.01 vs. wild type under the same treatment, ^#^ P<0.01 vs. *ena1Δ* under the same treatment, n = 3.

High Na^+^ concentration is one of the factors for toxicity of salt stress [4]. We asked if the salt sensitivity of *bdf1Δ* is due to Na^+^ toxicity. The intracellular Na^+^ concentration was detected by atomic absorption spectrophotometry. ENA1, which encodes a Na^+^-ATPase that pumps the excess intracellular Na^+^ out of the cells [30], [31], was used as a positive control. When cells were treated with NaCl, the Na+ concentration in *ena1Δ* was higher (p<0.01) than that in wild type (Fig. 1). It was also higher (p<0.01) than that in *bdf1Δ* either in the presence or absence of NaCl. Since both the *bdf1Δ* and *ena1Δ* mutants were sensitive to 0.5 mol.L^−1^ NaCl [15], these results suggested that the salt sensitivity of *bdf1Δ* was not caused by high intracellular Na^+^ concentration.

### 2. *HAL2* overexpression in *bdf1Δ* increased salt stress resistance and *bdf1ΔHal2*Δ double deletion strain was more sensitive to salt stress

**Figure 2:**
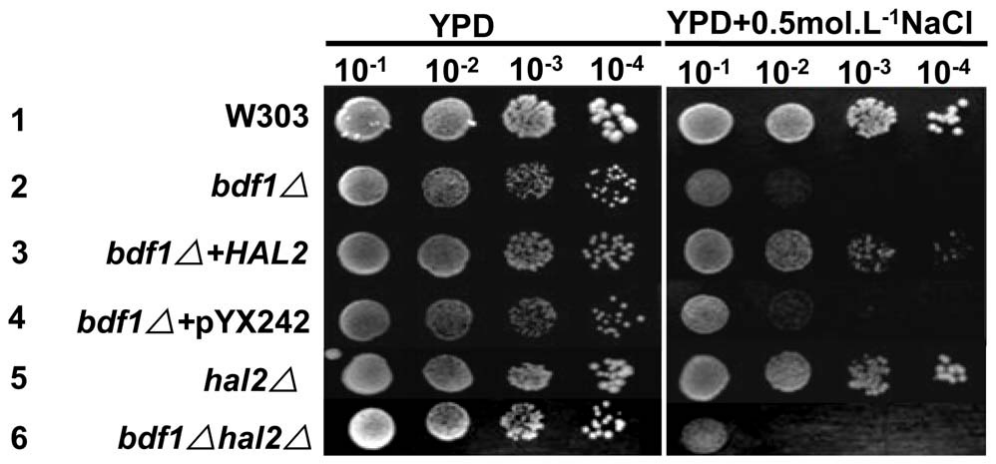
Overexpression of *HAL2* in *bdf1Δ* recovered its resistance to NaCl. 5 µl aliquots of 10-fold serial dilutions of the mid-log phase cultures were spotted onto YPD plates and incubated at 30°C for 3 d.

The nucleotidase Hal2p is a detoxifying enzyme and a target of high concentration of Na^+^, which competitively replaces Mg^2+^ in the active core of Hal2p [32]. To confirm whether Hal2p affects the salt sensitivity of *BDF1* deletion, *Hal2* deletion and *HAL2* overexpression strains were constructed (Table 1). Overexpression of *HAL2* in *bdf1Δ* significantly increased the resistance to salt stress (Fig. 2, line 3). *HAL2* deletion alone had no significant effect on salt sensitivity in YPD medium (Fig. 2, line 5). The *bdf1ΔHal2*Δ double deletion, however, was more sensitive to Na^+^ stress (Fig. 2, line 6) than *bdf1Δ* single deletion. This result suggests salt sensitivity of the *bdf1Δ* can be ameliorated by overexpression of *HAL2* and the *BDF1*- and *HAL2*-involved salt stress responses may be different.

### 3. Deletion of BDF1 reduced the expression level of *HAL2*

**Figure 3:**
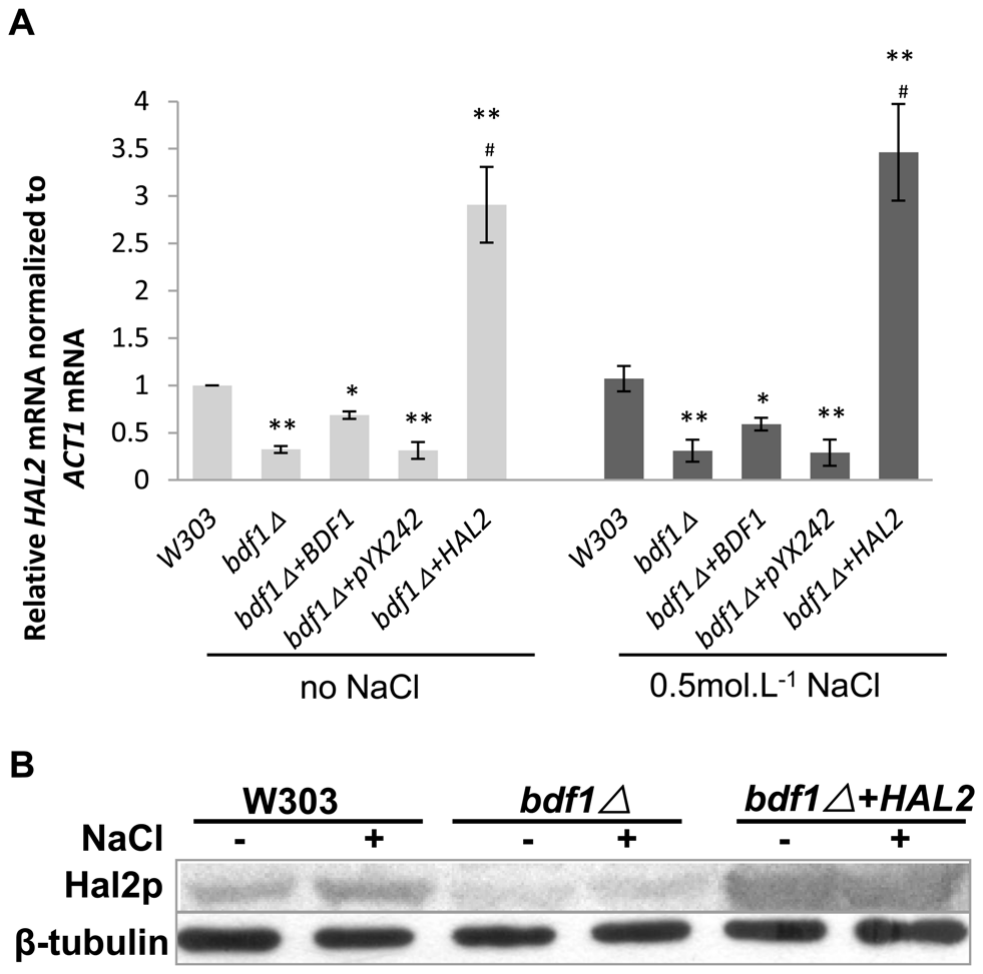
Deletion of *BDF1* reduced *HAL2* expression at both mRNA and protein levels. The mid-log phase cultures in YPD at 30°C were incubated for 45 min with or without 0.5 mol.L^−1^ NaCl. (A) Relative fold-changes of *HAL2* mRNA were calculated against the wild type without NaCl treatment. Error bars denote standard deviation (SD). **P<0.01 vs. wild type under the same treatment, ^#^ P<0.01 vs. *bdf1Δ* under the same treatment, n = 3. (B) The Hal2p protein level was analyzed with whole cell protein by Western blot. β-tubulin was used as control. The polyclonal Hal2p antibody and anti-β-tubulin antisera were used in Western blot analysis.

To confirm if *HAL2* is involved in the *bdf1Δ*-induced salt sensitivity,the expression level of *HAL2* was detected by RT-qPCR. As shown in Fig. 3A, the *HAL2* mRNA level in the *bdf1Δ* mutant was about two folds lower than that in wild type (p<0.01). No significant changes in *HAL2* mRNA levels were observed in the *bdf1Δ* strain between cells with and without NaCl (0.5 mol.L^−1^) treatment. When *BDF1* was reintroduced into *bdf1Δ* strain using a 2 µ plasmid, *HAL2* mRNA level was partially recovered, suggesting that *BDF1* affects the *HAL2* expression (Fig. 3A). Spot assay showed that *bdf1Δ*+*BDF1* is less resistant to salt stress than the wild type (Fig. S1), suggesting that the expression level of the 2 µ plasmid encoded BDF1 is less than the endogenous *BDF1*. This is most likely the reason that the *HAL2* mRNA level was only partially recovered in *bdf1Δ*+*BDF1*. The *HAL2* mRNA level in *bdf1Δ*+*HAL2* was about 9 folds higher than that in *bdf1Δ* (p<0.01) when cells were treated with or without NaCl. A similar pattern was observed at protein level by Western blot analysis (Fig. 3B). After *BDF1* was deleted, *HAL2* protein level was reduced to ∼30% of that in wild type (p<0.01). The *HAL2* protein level in *bdf1Δ*+*HAL2* was about 9 folds higher than that in *bdf1Δ*. This indicated *BDF1* could affect the *HAL2* expression at mRNA level and protein level.

### 4. Bdf1p does not regulate *HAL2* expression directly

**Figure 4:**
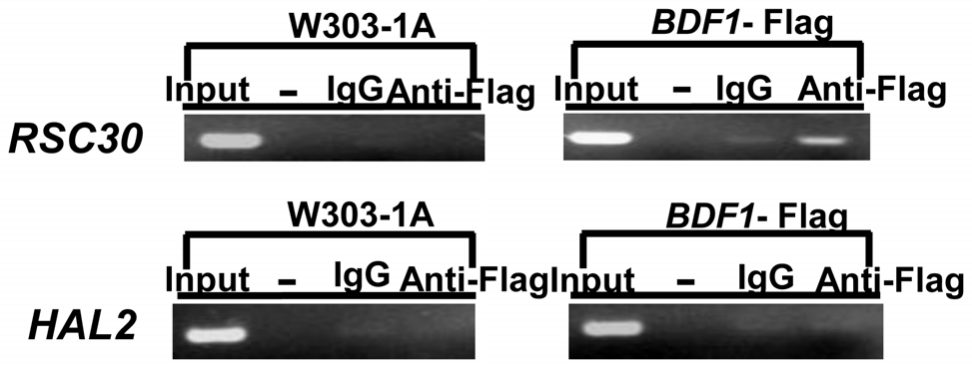
Bdf1p regulated *HAL2* expression indirectly. The anti-Flag antibody was used for Bdf1p precipitation and IgG was used as a negative control. The promoter regions probed by ChIP correspond to nucleotides −200 to −1. Error bars denote standard deviation (SD). *P<0.05, **P<0.01 vs. wild type under the same treatment, n = 3.

Chromatin immunoprecipitation (ChIP) was used to detect how *HAL2* expression is regulated by Bdf1p. The promoter regions probed by ChIP correspond to nucleotides −200 to −1 relative to the translation start sites and these regions were used for PCR detection. The promoter of RSC30, which is directly bound by Bdf1p [33], was used as a positive control. Fig. 4 showed that the promoter region of RSC30 but not *HAL2* was detected in Bdf1p immunoprecipitation. This result, together with that of *HAL2* expression (Fig. 3), suggests that the *HAL2* expression is positively regulated by Bdf1p but in an indirect manner. We speculate that other transcription factor(s) are involved to mediate the interaction between Bdf1p and *HAL2* expression.

### 5. Salt sensitivity of *bdf1Δ* was not due to high level of intracellular pAp or the lack of methionine

**Figure 5:**
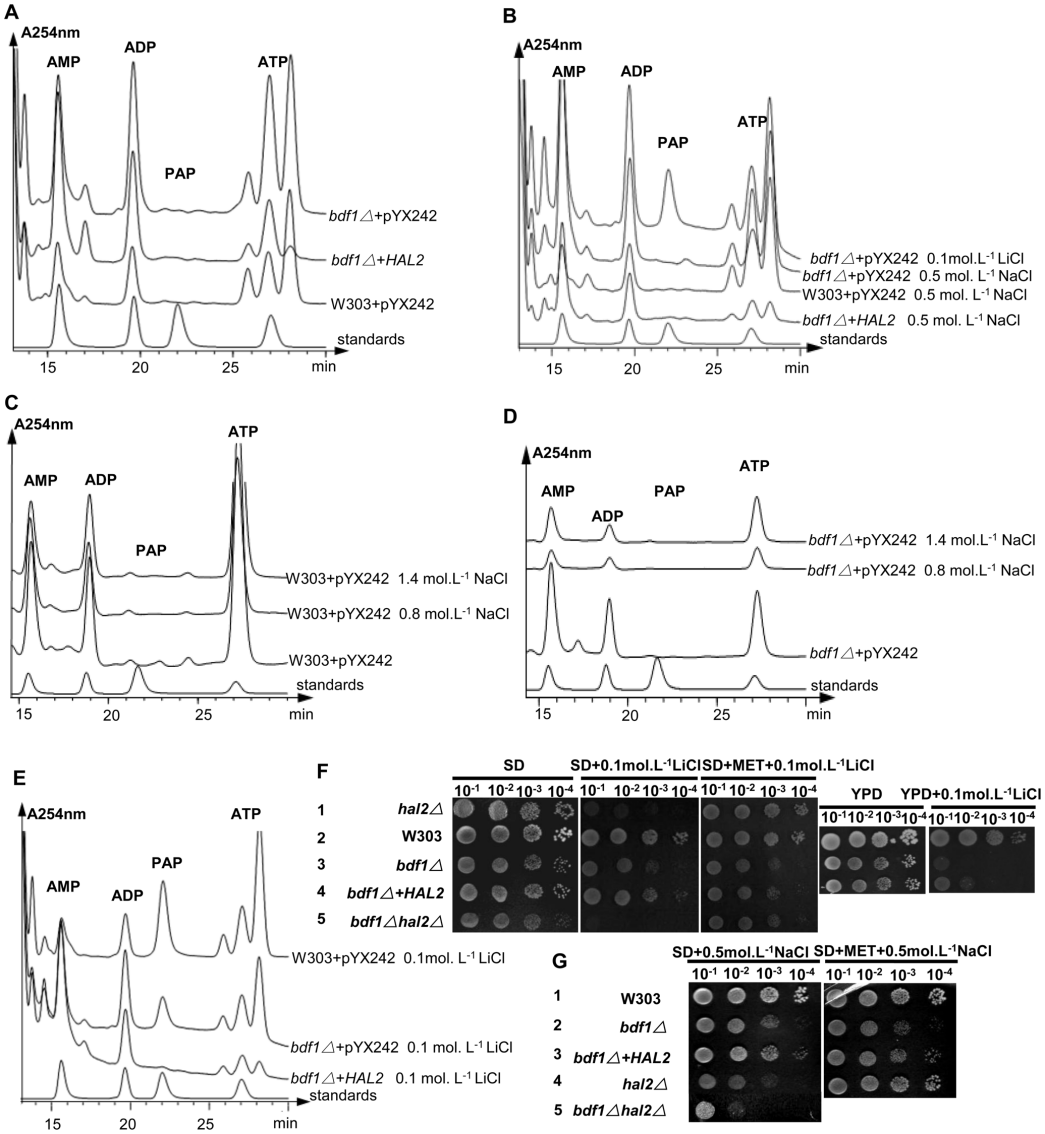
Salt sensitivity of *bdf1Δ* was not due to intracellular pAp or the lack of methionine. Cells grown to OD_600_ = 0.2∼0.4 in SD medium were incubated at 30°C without or with different concentrations of NaCl for 4 h, or 0.1 mol.L^−1^ LiCl for 2 h. The intracellular pAp concentration was determined as described in Materials and Methods (A–E). 5 ul aliquots of 10-fold serial dilutions of the mid-log phase cultures were spotted onto plates and incubated at 30°C for 3 d (F, G).

Hal2p, a detoxifying enzyme, converts toxic 3′-phosphoadenosine-5′-phosphate (pAp) into nontoxic AMP and Pi in the process of sulfate assimilation to methionine [34]. Methionine supplementation or *HAL2* overexpression could improve salt tolerance [32]. Although there was no Na^+^ accumulation in *bdf1Δ* strain (Fig. 1), *BDF1* deletion caused a decreased level of *HAL2* expression (Fig. 3A). To see if the salt sensitivity of *bdf1Δ* is caused by pAp accumulation the intracellular pAp concentrations was measured by HPLC. Unlike the RS-16 strain of S cerevisiae, in which pAp accumulation was detectable when treated with NaCl (0.6 mol.L^−1^ or higher) [16], [34], no pAp was detected in wild type (W303 background) or its derived stains either without (Fig. 5A) or with (Fig. 5B) NaCl treatment, even when NaCl concentration was increased to 1.4 mol.L^−1^ (Fig. 5C, D). However, when cells were treated with 0.1 mol.L^−1^ of LiCl, accumulation of pAp was detected in wild type as previously reported [35] as well as in *bdf1Δ* (Fig. 5E). Overexpression of *HAL2* diminished the pAp accumulation in *bdf1Δ* (Fig. 5E). Spot assay showed that overexpression of *HAL2* in *bdf1Δ* partially suppressed the sensitivity to Li^+^ or Na^+^ in both YPD and SD media (Fig. 2, Lines 2 and 3 and Figs. 5F, lines 3 and 4, 5G, Lines 2 and 3). As reported previously [32], addition of methionine (20 mg.L^−1^) recovered the Na^+^ and Li^+^ resistance of *hal2*Δ. Addition of methionine did not, however, recover the salt sensitivity of *bdf1Δ* (Figs. 5F, lines 1 and 3, 5G, Lines 1 and 3). These results suggest that the high level of Hal2p was required for increasing the resistance of *bdf1Δ* to both sodium and lithium salt stress. It also suggests that the sensitivity of *bdf1Δ* to Na^+^ or Li^+^ stress was not due to the accumulation of pAp or lack of methionine.

In addition to its detoxifying function, Hal2p is involved in amino acid metabolism [34]. The *hal2*Δ exhibited Na^+^ sensitivity on medium lacking amino acids app:ds:lack(Fig. 5G, Line 4) but not on medium rich in amino acids (Fig. 2, Line 5). Meanwhile, *bdf1Δhal2*Δ double mutant showed enhanced sensitivity to Na^+^ compared to either single mutant (Fig. 5G, Lines 2, 4, 5 and Fig. S2). These results suggest that the pathway of Bdf1p-involved Na^+^ stress response could be different from that of Hal2p. This notion is supported by the fact that amino acids can rescue the salt stress of *hal2* but not bdf1 mutants.

### 6. *HAL2* overexpression reduced ROS accumulation and partially recovered mitochondrial function of *bdf1Δ*

It has been observed that salt stress in *bdf1Δ* caused an increase of ROS and decrease of Δ*φ* [14], both are markers of apoptotic cell death. The *bdf1Δ* grows slowly in nonfermentable carbon sources, such as glycerol, due to its mitochondrial respiratory deficiency [36].

Because overexpression of *HAL2* enhanced the salt resistance of *bdf1Δ* (Fig. 2, Line 3, Fig. 5G, Line 3), ROS levels and mitochondrial membrane potential (Δ*φ*) were measured [37], [38], to see if *HAL2* overexpression causes any changes of ROS level and mitochondrial membrane potential.

**Figure 6:**
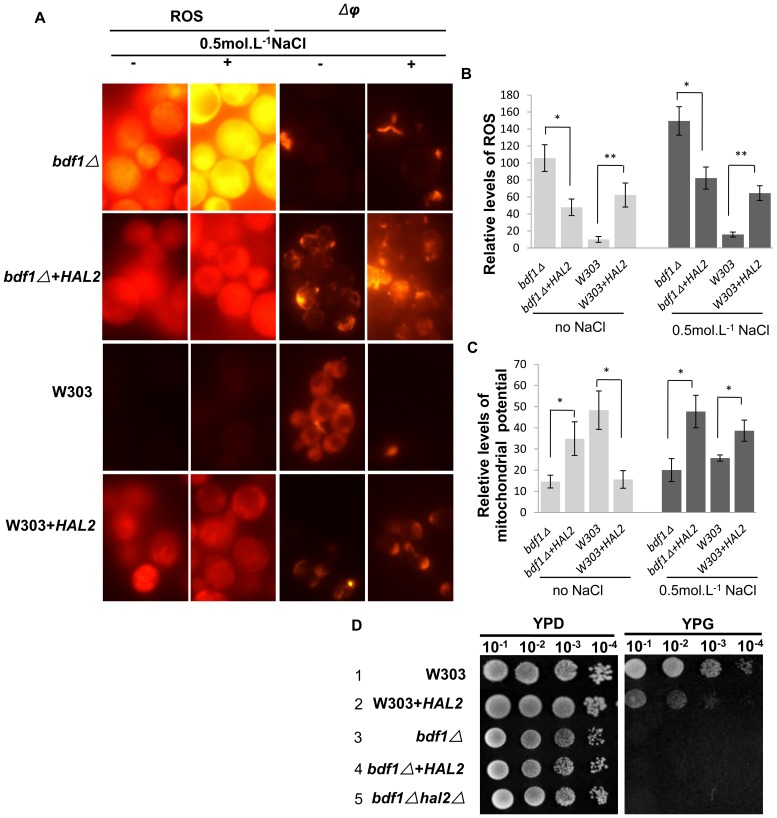
*HAL2* overexpression affected ROS accumulation and partially affected the mitochondrial function. Mid-log phase cells were incubated for 45 min with or without 0.5 mol.L^−1^ NaCl. ROS were detected by dihydrorhodamine 123. Mitochondrial membrane potential was measured with JC-1 (A) The ROS (B) and *Δ*φ (C) fluorescence values were quantified as the relative fluorescence intensity per strain by ImageJ software and averaged from ∼50 cells. Error bars denote standard deviation (SD). *P<0.05, **P<0.01 vs. strains without *HAL2* overexpression under the same treatment, n = 4. (D) 5 ul aliquots of 10-fold serial dilutions of the mid-log phage cultures were spotted onto YPG plates and incubated at 30°C for 3 d.

Our result showed that overexpression of *HAL2* in *bdf1Δ* reduced the ROS level both in the absence (0.55 fold) (p<0.05) and in the presence (0.45 fold) (p<0.05) of NaCl (Fig. 6A, B). It also enhanced the Δ*φ* (1.39 folds without NaCl, p<0.05; or 1.38 folds with NaCl, p<0.05) (Fig. 6A, C). These results suggest that *HAL2* was indeed involved in the removal of ROS and in augment of Δ*φ* in *bdf1Δ* cells. Surprisingly, overexpression of *HAL2* in wild type induced ROS production (5.20 folds without NaCl, p<0.01; or 3.08 folds with NaCl, p<0.01) (Fig. 6A, B). *HAL2* overexpression also decreased mitochondrial membrane potential (Δ*φ*) (0.67 fold without NaCl, p<0.05; or 0.50 fold with NaCl, p<0.05) (Fig. 6A, C). Unlike the wild type, *bdf1Δ* did not grow normally on glycerol medium (YPG) even with *HAL2* overexpression (Fig. 6D, Lines 3, 4) and *HAL2* over-expression caused the wild type to grow slower (Fig. 6D, Lines 1, 2), suggesting that Hal2p helped to reduce ROS accumulation and recover the mitochondrial function but not the respiratory deficiency in *bdf1Δ*. The excess amount of Hal2p in wild type may cause damage to cells.

### 7. Autophagy was defective in *bdf1Δ* and stimulated by *HAL2* overexpression

Autophagy is an intracellular catabolic process through autophagosomes to degrade the cytosolic components [39]–[41]. This process counteracts with internal and external stresses and alters the cell metabolic equilibrium [39]. We speculate that autophagy plays a role in the Hal2p involved ROS removal. To test this possibility, we tagged the Atg8p, which localizes on the inner membrane of autophagosome [42], [43], with GFP at its N-terminus (GFP-*ATG8*) as a marker to detect the autophagy level [43], [44]. The autophagosomes were also observed using a bright-field light microscope.

**Figure 7:**
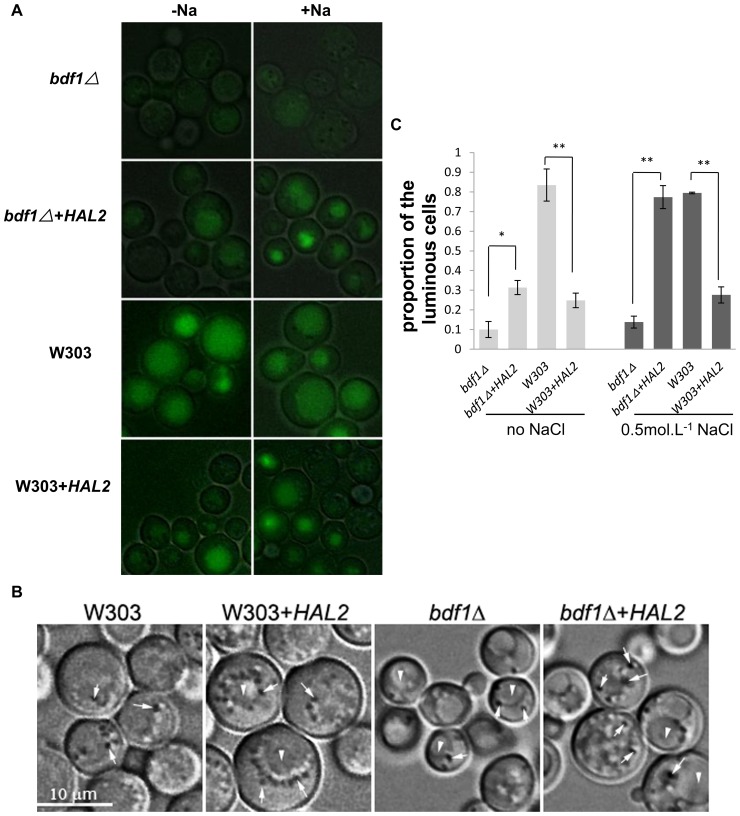
*BDF1* was required for autophagy and *HAL2* stimulated autophagy. Yeast cells of OD600>1.5 were collected from SC medium and incubated for 45 min with (+) or without (−) 0.5 mol.L^−1^ NaCl. (A) Cells were transformed with the pRS316-GFP-*ATG8*. The GFP-*ATG8* fluorescence images were merged with bright field images to show the outlines of the cells. (B) The autophagosomes were observed by a bright-field light microscope. Arrows indicate autophagosomes; arrowheads indicate vacuoles. (C) Cells with fluorescence were measured from about 200 cells. Error bars denote standard deviation (SD). *P<0.05, **P<0.01 vs. strains without *HAL2* overexpression under the same treatment, n = 3.

We first checked the autofluorescence background of strains with no GFP tag. All strains showed a similar but constant autofluorescence with or without NaCl treatment (data not shown). The GFP-*ATG8* of *bdf1Δ* cells exhibited very weak GFP fluorescence regardless of NaCl treatment (Fig. 7A). Overexpression of *HAL2* in *bdf1Δ* significantly increased the fluorescence intensity of GFP-*ATG8*. Proportion of cells with fluorescence was also increased (p<0.05) either with or without NaCl treatment (Fig. 7C). Overexpression of *HAL2* in wild type also caused an increase in fluorescence intensity, either with or without NaCl treatment (Fig. 7A) although the proportion of cells with GFP fluorescence (Fig. 7C) reduced. This reduction could be due to cell death induced by autophagy. Overall, Na^+^ stress enhanced autophagy fluorescence in all the strains (Fig. 7A). We also observed more autophagosomes around the vacuoles in both *bdf1Δ* and wild type strains with *HAL2* overexpression (Fig. 7B). These results suggest that *HAL2* overexpression could stimulate autophagy.

Taken together, we believed that *HAL2* overexpression reduced ROS accumulation and partially recovered the mitochondrial function in the *bdf1Δ*. In contrast, overexpression of *HAL2* in wild type appeared to damage the respiration of mitochondria possibly by the excessive autophagy and recycling of cytoplasmic contents, such as mitochondria, into the vacuole. The excessive autophagy may cause stress itself, therefore, increasing the ROS level in wild type cells.

## Discussion

### 1. Overexpression of *HAL2* reduces saltsensitivity of *bdf1Δ* possibly via removal of ROS by *HAL2*-stimulated autophagy

High intracellular Na^+^ concentration is one of the most important factors to salt stress in yeast. Our data showed, however, that the concentrations of Na^+^ in wild type and *bdf1Δ* were similar and both were lower than that of *ena1Δ* (Fig. 1). Ena1p is the Na^+^-ATPase that pumps the excess intracellular Na^+^ out of the cells [30]. This indicates the sodium discharge pump is functional in *bdf1Δ* and Na^+^ toxicity is not the main reason of *bdf1Δ* salt sensitivity.

To investigate the possible reasons of *bdf1Δ* salt sensitivity, we identified several genes that can recover salt sensitivity of *bdf1Δ*, and *HAL2* is one of them. Overexpression of *HAL2* in *bdf1Δ* recovered its sensitivity to Na^+^ in both rich (Fig. 2, Line 3) and minimal media (Fig. 5G, Line 3).

We previously reported that the *BDF1* deletion aggravated apoptosis under Na^+^ stress due to mitochondrial dysfunction [15]. Overexpression of *HAL2* restrained *bdf1Δ* Na^+^ sensitivity (Fig. 2, Fig. 5G). Therefore, the mitochondria function index ROS and Δ*φ* as well as cells growth on a non-fermentable carbon source were examined. Overexpression of *HAL2* in *bdf1Δ* increased the Δ*φ* and decreased the ROS level (Fig. 6). The opposite effect, however, occurred when *HAL2* was overexpressed in wild type. Cells (W303+*HAL2*, Table 1) displayed ROS accumulation, Δ*φ* decrease and growth decrease on glycerol containing medium (Fig. 6). These data indicate that *HAL2* overexpression recovered part of the mitochondria functions in *bdf1Δ* mutant but may induce harmful effects in the wild type cells.

Comparison of GSH/GSSG ratio between WT+*HAL2* and *bdf1Δ*+*HAL2* after salt treatment showed that the GSH/GSSG ratio of *bdf1Δ*+*HAL2* is much higher than that of WT+*HAL2* (Chen and Bao, unpublished data). This result further suggests that *HAL2* overexpression may reduce the ROS level of *bdf1Δ* under salt stress.

Under unfavorable conditions like nutrient deprivation, yeast cells can make molecules recycled by a non-selective autophagy process for their own cytosolic components with the autophagosomes, which are visible under the bright-field microscope [39], [40], [45]. Since Hal2p overexpression caused both positive and negative impacts on the mitochondria function (Fig. 6), we app:ds:conjecturespeculate that the non-selective degradation process in autophagy may play a role in this process.

Defective autophagy with high level of ROS and mitochondria dysfunction [46] was observed in *bdf1Δ* (Fig. 6, Fig. 7). Overexpression of *HAL2* in *bdf1Δ* induced autophagy, elevated the fluorescence intensity of GFP-Atg8p, partially restored mitochondrial function and Na^+^ resistance. This led us to conclude that Hal2p overexpression stimulates autophagy and the defect of autophagy in *bdf1Δ* could be one of the causes for Na^+^ sensitivity. We speculated that the autophagy induced by *HAL2* overexpression is probably related to amino acids imbalance. The overexpression of *HAL2*, a participator of methionine biosynthesis, probably mediates some amino acids starvation, therefore inducing autophagy and recovering the salt stress sensitivity of *bdf1Δ*. This is further confirmed by our microarray analysis, which showed that many genes related to amino acids metabolism and iron acquisition were upregulated, when *HAL2* was overexpressed in *bdf1Δ* (Chen and Bao, unpublished data).

### 2. Hal2p and Bdf1p respond to salt stress via different mechanisms

Methionine supplementation rescued the growth of *hal2*Δ mutants but not *bdf1Δ* mutants, suggesting the differences in Na^+^ stress response between *HAL2* and *BDF1*. In addition, *HAL2* overexpression recovered the cell growth of *bdf1Δ* under either Li^+^ or Na^+^ stress (Fig. 5F, G), but no significant difference of pAp accumulation was observed under Na^+^ stress suggesting that the mechanisms mediated by Bdf1p and Hal2p in response to these two salts are different.

*HAL2* deficiency caused Na^+^ sensitivity but only on medium lacking amino acid (Fig. 5G, Line 4), possibly because Hal2p is related to amino acid metabolism [34]. The *bdf1Δhal2*Δ double deletion mutant showed enhanced sensitivity to NaCl compared to the two single deletion mutants (Fig. 5G, Lines 2, 4, 5). This result further suggests that the mechanism of Hal2p-mediated in Na^+^ stress response is different from that of Bdf1p-mediated.

One interesting phenomenon we observed is that unlike the S. cerevisiae RS-16 background strain [16], [34], no pAp was detected in W303 background strains even under a high level of NaCl stress (Fig. 5A–D). Under Li^+^ stress, there was a high level of pAp accumulation in both W303 wild type and the derived *bdf1Δ* and pAp accumulation in *bdf1Δ* cells under Li^+^ stress could be diminished by *HAL2* overexpression (Fig. 5E). This indicated that pAp accumulation was not related to Na^+^ salt sensitivity of *bdf1Δ*. Methionine supplementation rescued the growth of *hal2*Δ mutants but not *bdf1Δ* mutants,

The difference in pAp accumulation between W303 and RS-16 is probably due to the different genotypes of the two strains. W303, but not RS-16, is ade1 mutant (Table 1) [16], [34]. As a result, the colony of W303 appears to be remarkably red, a sign of red pigment accumulation due to ade1 defect.

## Supporting Information

Figure S1 **Expression of** ***BDF1*** **using a 2 μ plasmid enhanced the salt resistance of** ***bdf1Δ*** **.** 5 µl aliquots of 10-fold serial dilutions of the mid-log phase cultures were spotted onto YPD plates with or without NaCl and incubated at 30°C for 3 d.
(TIF)

Figure S2 ***bdf1Δhal2Δ*** **double deletion is more sensitive to salt stress.** 5 µl aliquots of 10-fold serial dilutions of the mid-log phase cultures were spotted onto YPD plates with or without NaCl and incubated at 30°C for 3 d.
(TIF)

Table S1 **Primers used in the current study.**
(DOCX)
