# Supplementary figures and images for: Hal2p Functions in Bdf1p-Involved Salt Stress Response in Saccharomyces cerevisiae
Source: PLoS One. 2013 Apr 17;8(4):e62110. doi: 10.1371/journal.pone.0062110 (PMC3629146; doi:10.1371/journal.pone.0062110)

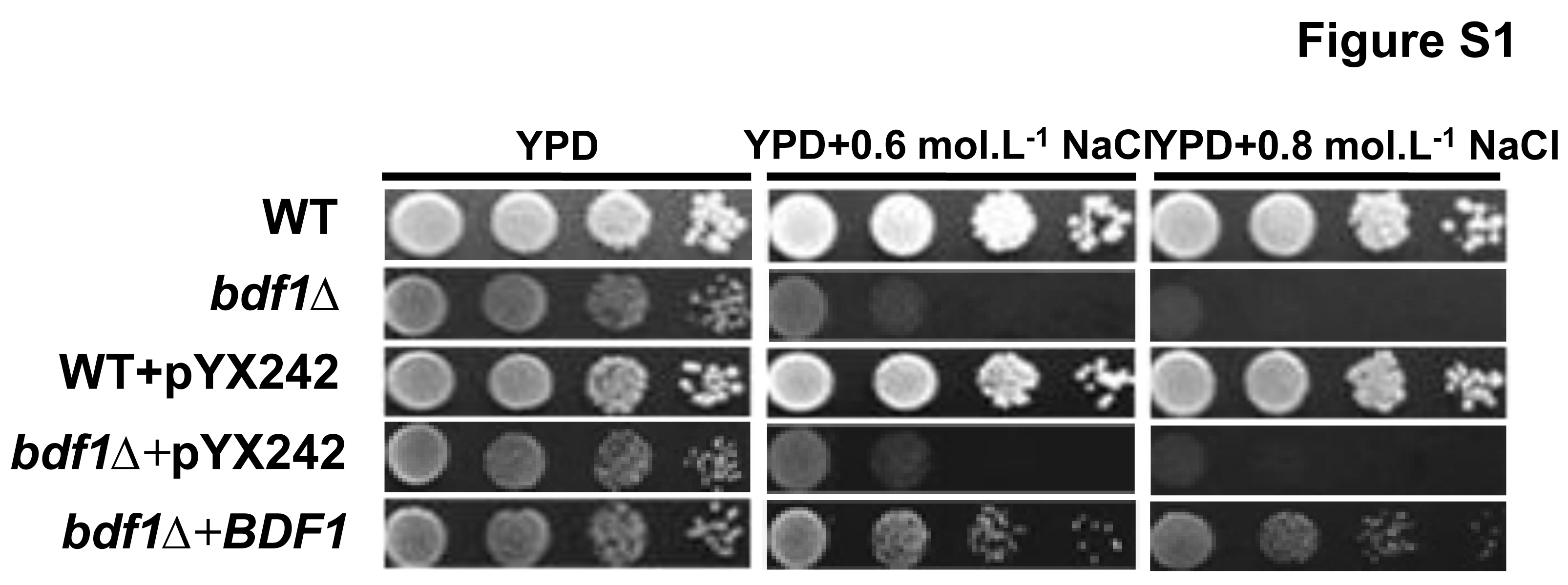

Supplement: Figure S1 — Expression of BDF1 using a 2 μ plasmid enhanced the salt resistance of bdf1Δ . 5 µl aliquots of 10-fold serial dilutions of the mid-log phase cultures were spotted onto YPD plates with or without NaCl and incubated at 30°C for 3 d. (TIF) [file pone.0062110.s001.tif]

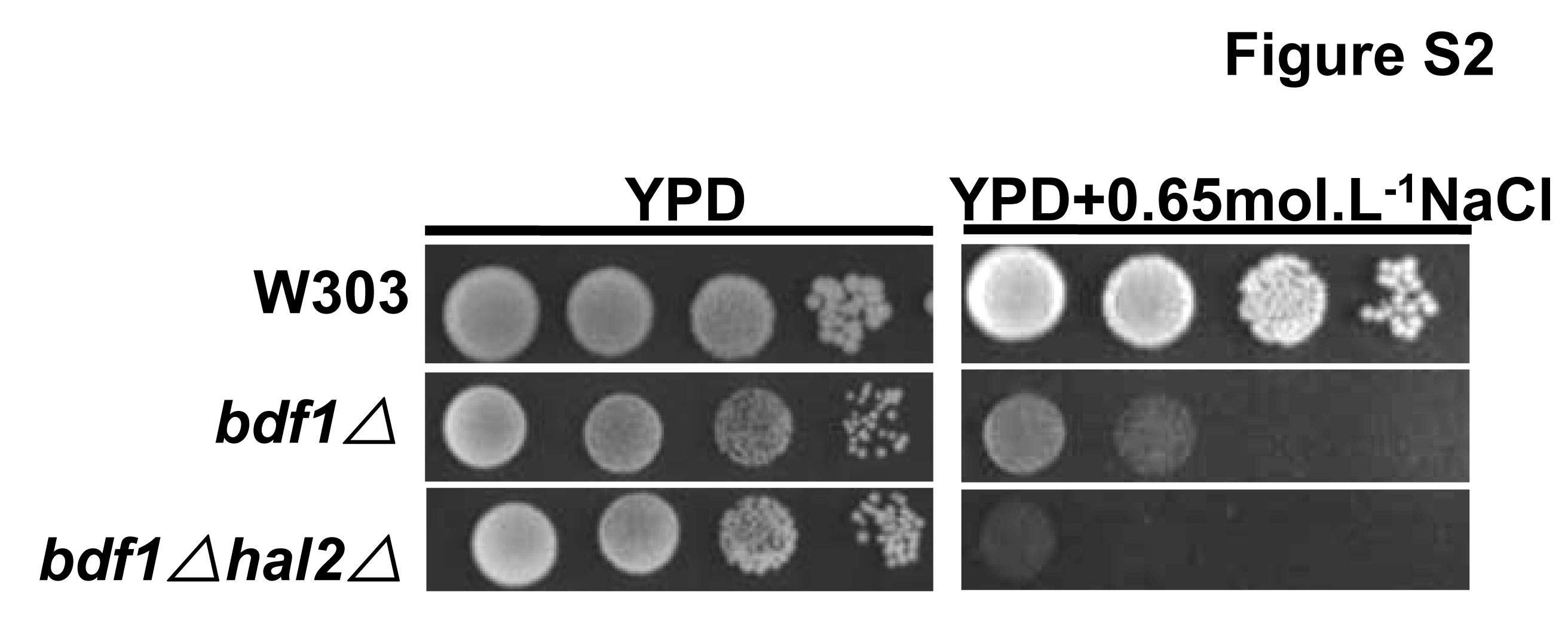

Supplement: Figure S2 — bdf1Δhal2Δ double deletion is more sensitive to salt stress. 5 µl aliquots of 10-fold serial dilutions of the mid-log phase cultures were spotted onto YPD plates with or without NaCl and incubated at 30°C for 3 d. (TIF) [file pone.0062110.s002.tif]
