# Supplementary material for: Hal2p Functions in Bdf1p-Involved Salt Stress Response in Saccharomyces cerevisiae
Source: PLoS One. 2013 Apr 17;8(4):e62110. doi: 10.1371/journal.pone.0062110 (PMC3629146; doi:10.1371/journal.pone.0062110)
Supplement: Table S1 — Primers used in the current study. (DOCX) [file pone.0062110.s003.docx]

**Supplemental material**

**Table S1 Primers used in the current study**

| Primers | Sequence (5’-3’)* | Description |
| --- | --- | --- |
| *HAL2*-L1 | GACATTTACATAGCCATCTAT | Deletion of *HAL2* |
| *HAL2*-L2 | GTCATCACCGAAACGCGCAGTAAAGTGCTGATGTCTTC | Deletion of *HAL2* |
| *HAL2*-L3 | GAAAATACCGCATCAGGAAAATAACATATATTTTACTTTATTGAT | Deletion of *HAL2* |
| *HAL2*-L4 | GTTCATTCGGTCAGAATCC | Deletion of *HAL2* |
| *HAL2-F* | CGTACCTAGGATGG CATTGGAAAGAGAATTA | Construction of Plasmid pYX242-*HAL2* |
| *HAL2-R* | GTCACCTAGGGGCGTTTCTTGACTGAATG | Construction of Plasmid pYX242-*HAL2* |
| *BDF1-*F, | CGTAGAATTCAT GACCGATATCACACCC | Construction of Plasmid pYX242-*BDF1* |
| *BDF1-*R | GTCAGAATTCCTCTTCTTCACTTTCGCT | Construction of Plasmid pYX242-*BDF1* |
| qPCR *HAL2-F* | GGTGCCTTCTATTCTCCATCTTC | qPCR |
| qPCR *HAL2-R* | AGTGTCCCTTTTCAACTCCCTC | qPCR |
| qPCR *ACT1-F* | ATGCAAACCGCTGCTCAA | qPCR |
| qPCR *ACT1-R* | AGTTTGGTCAATACCGGCAGA | qPCR |
| *RSC30* (from -614 to -394) -F | TGTCCAGAGATGCGAATGA | CHIP |
| *RSC30* (from -614 to -394) -R | ACTAATGCTGCGCTAACCAGA | CHIP |
| *HAL2* (from -215 to ^-1^) -F | CATATTTTGACATTTACATAGC | CHIP |
| *HAL2* (from -215 to ^-1^) -R | AGTAAAGT GCTGATGTCTTC | CHIP |
| GFP-F | CGGGATCCATGTCTAAAGGTGAAGAATTAT | Construction of Plasmid pRS316-*GFP-ATG8* |
| GFP+ATG-M –reverse | CAGACTTAAATGTAGACTTCATTTTGTACAATTCATCCATACC | Construction of Plasmid pRS316-*GFP-ATG8* |
| GFP+ATG-M | GGTATGGATGAATTGTACAAAATGAAGTCTACATTTAAGTCTG | Construction of Plasmid pRS316-*GFP-ATG8* |
| ATG8-R | CCAAGCTTCTACCTGCCAAATGTA | Construction of Plasmid pRS316-*GFP-ATG8* |

*The sequences of the restriction sites are underlined.
